# Adjunctive Pentoxifylline Enhances Clinical Remission and Reduces Inflammatory Biomarkers in Mild-to-Moderate Ulcerative Colitis: A Randomized Double-Blind Placebo-Controlled Pilot Trial

**DOI:** 10.3390/ph19040552

**Published:** 2026-03-30

**Authors:** Mohannad O. Khrieba, Furqan M. Abdulelah, Amal Mohammed Badawoud, Eman Hamza, Reham A. Al-Dhelaan, Tarek I. Ahmed, Ahmed G. Abdelhameed, Nora Elshorbagi, Doaa A. El-Hanafy, Nashwa Eltantawy, Muhammed M. Salahuddin, Noha M. Elkhodary, Kholoud H. Radwan, Khaled Abo Bakr Khalaf Ali, Abeer A. El-Sayed, Marwa Kamal

**Affiliations:** 1 Pharmacy Practice Department, Faculty of Pharmacy, Horus University-Egypt, New Damietta 34517, Egypt; mkhrieba@horus.edu.eg; 2 Pharmacy Practice Department, Faculty of Pharmacy, East Port Said National University, Port Said 42511, Egypt; 3 Clinical Pharmacy Department, College of Pharmacy, Al-Naji University, Baghdad 10015, Iraq; 4 Department of Pharmacy Practice, College of Pharmacy, Princess Nourah Bint Abdulrahman University, Riyadh 11671, Saudi Arabia; 5 Department of Biochemistry, College of Medicine, Imam Mohammad Ibn Saud Islamic University (IMSIU), Riyadh 13317, Saudi Arabia; 6 Internal Medicine Department, Faculty of Medicine, Fayoum University, Fayoum 63514, Egypt; 7 Pharmacology and Toxicology Department, Faculty of Pharmacy, Mansoura University, Mansoura 35516, Egypt; 8 Pharmacology and Biochemistry Department, Faculty of Pharmacy, Horus University-Egypt, New Damietta 34517, Egypt; 9 Pharmacology and Toxicology Department, Faculty of Pharmacy, Sinai University, East Kantara Branch, Ismailia 41636, Egypt; 10 Clinical Pathology Department, Faculty of Medicine, Mansoura University, Mansoura 35516, Egypt; 11 Clinical Pharmacy and Pharmacy Practice Department, Faculty of Pharmacy, Egyptian Russian University, Badr City 11829, Cairo, Egypt; 12 Department of Clinical Pharmacy, Faculty of Pharmacy, Kafrelsheikh University, Kafr El-Sheikh 33516, Egypt; 13 Biochemistry Department, Horus University-Egypt, New Damietta 34517, Egypt; 14 Tropical Medicine and Gastroenterology Department, Faculty of Medicine, Assiut University, Assiut 71515, Egypt; 15 Department of Pharmacy Practice, Faculty of Pharmacy, Arish Branch, Sinai University, Arish 45511, Egypt; 16 Clinical Pharmacy Department, Faculty of Pharmacy, Fayoum University, Fayoum 63514, Egypt

**Keywords:** ulcerative colitis, PTX, TNF-α, ESR, calprotectin, IBDQ-32

## Abstract

**Background**: Despite mesalamine’s efficacy in mild-to-moderate ulcerative colitis (UC), many patients fail to achieve complete clinical or biochemical remission. Pentoxifylline (PTX) may act as an adjunct therapy by modulating cytokine production and oxidative stress. **Aim**: To evaluate the therapeutic effect of adding PTX in patients with UC. **Methods**: In this randomized, double-blind, placebo-controlled pilot study, 60 patients with UC were assigned to mesalamine plus placebo (Group 1) or mesalamine plus PTX 400 mg BID (Group 2) for 24 weeks. The primary outcome was changes in the partial Mayo score (PMS). Clinical remission was defined as PMS ≤ 2 with no subscore > 1; clinical response as a reduction in PMS ≥ 2 points. Quality of life (QoL) was measured using the Inflammatory Bowel Disease Questionnaire (IBDQ-32). Serum TNF-α, fecal calprotectin, and erythrocyte sedimentation rate (ESR) were assessed. Analyses were performed using intention-to-treat (ITT) and per-protocol (PP) approaches. Subgroup analyses stratified by prior mesalamine exposure, and multivariable regression adjusted for age, sex, disease duration, smoking, and disease extent. **Results**: PTX significantly improved PMS compared to placebo in both ITT and PP analyses. Clinical response and remission rates were higher with PTX. IBDQ-32 scores increased, and TNF-α, calprotectin, and ESR decreased significantly more with PTX. Improvements were consistent across mesalamine-naïve and experienced patients. Multivariable regression confirmed that these effects were independent of demographic or disease-related confounders. **Conclusions**: Adjunctive PTX significantly enhanced clinical outcomes, reduced inflammation, and improved QoL in UC patients, supporting its potential as an effective add-on therapy to mesalamine.

## 1. Introduction

Ulcerative colitis (UC) is a persistent, idiopathic form of inflammatory bowel disease (IBD) marked by uninterrupted inflammation of the colon’s mucosal lining, usually starting at the rectum and progressing proximally in a continuous manner [1,2]. It manifests clinically with recurrent episodes of abdominal pain, bloody diarrhea, urgency, tenesmus, and weight loss. The precise cause of UC is still unknown; however, it is thought to arise from a multifactorial interaction involving genetic susceptibility, immune system dysregulation, disturbances in gut microbiota composition, and various environmental factors [3,4].

The pathological hallmark of UC is an exaggerated mucosal immune response characterized by the increased level of inflammatory cytokines, notably tumor necrosis factor-alpha (TNF-α), interleukin-1β (IL-1β), and IL-6 [5]. These mediators drive the recruitment of inflammatory cells, disrupt the intestinal barrier, and sustain chronic inflammation. Furthermore, biomarkers such as C-reactive protein (CRP), fecal calprotectin, and erythrocyte sedimentation rate (ESR) are typically elevated in active disease, correlating closely with the degree of mucosal inflammation [6].

Mesalamine (5-aminosalicylic acid) remains the first-line therapy for inducing and maintaining remission in mild-to-moderate UC and exerts its effects primarily via local inhibition of cyclooxygenase and lipoxygenase pathways, as well as through scavenging reactive oxygen species [7]. However, a large number of patients fail to achieve complete remission or experience relapse despite optimal mesalamine therapy, necessitating the exploration of adjunctive or alternative treatment strategies that can modulate the inflammatory cascade more effectively while maintaining a favorable safety profile [8].

Pentoxifylline (PTX), a methylxanthine derivative and non-selective phosphodiesterase inhibitor, has been extensively studied for its anti-inflammatory and immunomodulatory actions [9]. It inhibits TNF-α production at the transcriptional level and downregulates several inflammatory cytokines, including IL-1β, IL-6, and interferon-γ [10]. Additionally, PTX improves microvascular circulation and reduces oxidative stress, making it a potentially valuable agent in inflammatory conditions such as IBD [11].

In preclinical studies, PTX has demonstrated significant therapeutic effects in experimental models of colitis [12,13,14,15]. In dextran sulfate sodium (DSS)-induced colitis, PTX administration reduced disease activity index, histologic damage, and colonic levels of TNF-α and malondialdehyde (MDA), a marker of lipid peroxidation [15]. Similarly, in trinitrobenzene sulfonic acid (TNBS)-induced colitis models, PTX treatment ameliorated colon inflammation, preserved mucosal architecture, and decreased myeloperoxidase (MPO) activity [13]. These effects were attributed to both the reduction in inflammatory pathways and the enhancement of antioxidant defenses such as superoxide dismutase (SOD) and glutathione peroxidase [16]. Clinically, PTX administration alleviated mucosal damage and decreased inflammatory mediators in patients with UC [10]. Recently, PTX administration alleviated extraintestinal manifestations of UC, such as pyoderma gangrenosum, in a case report [17].

Collectively, these findings provide a strong rationale for the clinical repurposing of PTX in the management of UC, especially in patients inadequately controlled on standard therapy. Despite the widespread use of mesalamine, many patients with UC do not achieve sustained remission, highlighting a critical need for adjunctive treatments [18]. PTX, with its dual action on pro-inflammatory cytokine suppression and oxidative stress reduction, could offer added benefit when used alongside mesalamine. Its oral route, affordability, and established safety in chronic use (e.g., peripheral vascular disease, diabetic nephropathy) further support its clinical applicability [19]. However, existing clinical evidence evaluating PTX in UC remains limited and is primarily restricted to preliminary or small-scale studies, with insufficient assessment of its additive benefit to standard therapy and limited evaluation of patient-centered outcomes such as quality of life and objective inflammatory biomarkers. Therefore, the present study was designed to address these gaps by investigating the efficacy of PTX as an adjunct to mesalamine in a controlled clinical setting, with a comprehensive evaluation of clinical disease activity, biochemical markers, and health-related quality of life.

We hypothesize that the addition of PTX to standard mesalamine therapy will result in superior clinical improvement, enhanced suppression of inflammatory markers, and better quality of life outcomes compared to mesalamine alone in patients with UC.

The primary aim of this study was to evaluate the efficacy and safety of PTX as an adjunctive therapy to mesalamine in patients with UC. Specific objectives included assessing its impact on clinical disease activity (partial Mayo score), inflammatory biomarkers (TNF-α, fecal calprotectin, and ESR), disease-specific quality of life (Inflammatory Bowel Disease Questionnaire—IBDQ), and tolerability and adverse events compared to standard therapy.

## 2. Results

### 2.1. Baseline Clinical, Demographic, and Laboratory Characteristics

The study included 60 individuals diagnosed with UC, who were randomly assigned to one of two treatment arms. The first group was administered mesalamine along with a placebo (n = 30), while the second group received a combination of mesalamine and PTX (n = 30). At the start of the trial, both groups demonstrated similar demographic profiles, clinical features, and laboratory parameters, with no statistically significant differences detected between them (Table 1).

The mean age of patients in the mesalamine group was 41.80 ± 8.48 years, compared to 39.27 ± 11.25 years in the PTX group (*p* = 0.329). The male-to-female ratio was similar in both groups (14/16 vs. 17/13; *p* = 0.438). There were no significant differences in weight (67.27 ± 6.08 vs. 69.87 ± 6.16 kg; *p* = 0.105) or body mass index (22.62 ± 1.51 vs. 22.86 ± 2.08 kg/m^2^; *p* = 0.599).

Liver function tests, including serum ALT and AST, were within normal ranges and showed no significant difference between groups (ALT: 28.83 ± 5.29 vs. 29.37 ± 3.10 IU/L, *p* = 0.636; AST: 31.90 ± 4.98 vs. 33.13 ± 6.86 IU/L, *p* = 0.421). Serum creatinine (SrCr) levels were also similar (0.94 ± 0.13 vs. 0.95 ± 0.10 mg/dL; *p* = 0.678), indicating preserved renal function in both groups. Hemoglobin and albumin levels were comparable (Hgb: 13.06 ± 1.07 vs. 12.74 ± 1.10 mg/dL, *p* = 0.253; Albumin: 4.35 [3.4–5.3] vs. 3.85 [3.49–5.6] g/dL, *p* = 0.950).

The mean platelet count did not differ significantly (195.7 ± 16.71 vs. 200.9 ± 13.43 × 10^3^/µL; *p* = 0.187). Disease duration was also similar between groups (0.7 [0–2.45] vs. 0.5 [0–1.72] years; *p* = 0.590). The number of treatment-naïve patients (11 vs. 14) and those with prior disease experience (19 vs. 16) showed no significant group differences (*p* = 0.614 and *p* = 0.686, respectively).

Regarding smoking status, seven patients in the mesalamine group and five in the PTX group were current smokers (*p* = 0.518). The disease extent was distributed as follows: proctitis (14 vs. 10), left-sided colitis (10 vs. 11), and extensive colitis (6 vs. 9), with no statistically significant difference in disease localization (*p* = 0.518).

### 2.2. Effect of Treatment on Clinical Markers

During the follow-up period, four patients were lost to follow-up in the mesalamine (placebo) group as they developed a severe disease state and shifted to adjuvant therapy with steroids, and three patients in the PTX group as they did not come back for the follow-up session. To preserve the integrity of randomization and reduce bias, ITT analysis was performed using the BOCF method for handling missing data. All randomized patients were included in the final analysis. All randomized patients were included in the final analysis, except that PMS was also analyzed using per-protocol analysis.

The clinical efficacy of the study medications was evaluated based on changes in the PMS, clinical response, and remission rates before and after treatment (Table 2).

At the start of the study, there was no statistically significant variation in PMS between the placebo group (median 5, IQR: 5–6) and the PTX group (median 5, IQR: 3–6). Following 24 weeks of therapy, both groups showed significant within-group improvements. In the placebo group, PMS decreased from 5 to 2 (IQR: 1–3; *p* < 0.001), while in the PTX group, PMS declined from 5 to 1 (IQR: 0–2; *p* < 0.001). These within-group differences were analyzed using the Wilcoxon signed-rank test.

The between-group comparison of PMS after treatment revealed significantly lower scores in the PTX group compared to the placebo group (*p* = 0.004), as determined by the Mann–Whitney U test, indicating enhanced clinical improvement with PTX.

The proportion of patients achieving a clinical response was significantly higher in the PTX group (76.66%) than in the placebo group (50%) (*p* = 0.032). Similarly, clinical remission was observed in 50% of patients receiving PTX versus 23.33% in the placebo group (*p* = 0.04). These between-group differences in categorical outcomes were assessed using the Chi-square test.

Effect size analysis demonstrated a consistent and clinically meaningful treatment effect. In the ITT analysis, the rank-biserial correlation indicated a moderate effect size (r_b_ = 0.408).

### 2.3. Effect of Treatment on Clinical Markers by Intention-to-Treat Analysis

Per-protocol analysis demonstrated a significant improvement in disease activity in both groups. In the placebo group, the median partial Mayo score (PMS) decreased from 5 (IQR: 4.5–6) at baseline to 2 (IQR: 1–3) after treatment (*p* < 0.0001). Similarly, in the PTX group, PMS was significantly reduced from 5 (IQR: 3–6) to 0.75 (IQR: 0–2) (*p* < 0.001).

Between-group comparison showed a significantly greater reduction in PMS in the PTX group compared to the placebo group (*p* = 0.0006). In terms of clinical outcomes, the PTX group achieved a higher response rate compared to the placebo group (85.18% vs. 57.7%, *p* = 0.02). Likewise, remission rates were significantly higher in the PTX group (55.55%) compared to the placebo group (26.9%) (*p* = 0.034) (Table 3).

Similarly, in the per-protocol (PP) analysis, the rank-biserial correlation also reflected a moderate effect size (r_b_ = 0.41).

### 2.4. Effect on Disease-Specific Quality of Life (IBDQ Subscales)

The IBDQ-32 was utilized to evaluate the patients’ quality of life, covering four subdomains: social, systemic, digestive, and emotional, in addition to the total IBDQ-32 score. Both treatment groups showed statistically significant improvements within their respective groups, as determined by the Wilcoxon signed-rank test. However, the PTX group demonstrated greater post-treatment improvements in most domains when compared to the mesalamine group as determined by Mann–Whitney U test (Table 4).

In the mesalamine group, the median score in the social domain increased significantly from 16 (IQR: 11–21) to 18.5 (IQR: 13–24.5) (*p* = 0.03), while in the PTX group, it improved from 15 (IQR: 11.75–20) to 23 (IQR: 19.75–28) (*p* < 0.001). The difference in post-treatment scores between the two groups was statistically significant in favor of the PTX group (*p* = 0.015; Mann–Whitney U test).

Similarly, in the systemic domain, scores improved from 15.5 (IQR: 9–19.25) to 18.5 (IQR: 15.75–21) in the mesalamine group (*p* = 0.003), and from 16.5 (IQR: 10.75–22.25) to 22.5 (IQR: 19.75–27) in the PTX group (*p* < 0.001). Post-treatment comparisons showed significantly better outcomes in the PTX group (*p* = 0.006).

In the digestive domain, both groups experienced marked improvement: the mesalamine group improved from 34 (IQR: 31.75–37) to 53.5 (IQR: 36–59) (*p* < 0.001), and the PTX group from 34 (IQR: 25–38) to 57 (IQR: 50.75–64) (*p* < 0.001), with a statistically significant advantage in the PTX group after treatment (*p* = 0.036).

For the emotional domain, the mesalamine group improved from 22 (IQR: 16.5–31) to 31 (IQR: 19–39) (*p* = 0.017), while the PTX group showed an increase from 27 (IQR: 15.5–38.5) to 36 (IQR: 24.75–42.25) (*p* < 0.001). However, the between-group difference after treatment was not statistically significant (*p* = 0.127).

Regarding the total IBDQ score, patients in the mesalamine group experienced an improvement from 85 (IQR: 71–102) to 121 (IQR: 104.5–146.3) (*p* < 0.001), whereas those in the PTX group improved from 100 (IQR: 74.75–109) to 135.5 (IQR: 119.3–149) (*p* < 0.001). The difference in total post-treatment scores between the two groups was statistically significant, favoring the PTX group (*p* = 0.036).

### 2.5. Effect on Inflammatory Serum and Fecal Parameters

The biological effects of PTX on inflammatory biomarkers were evaluated by measuring serum levels of TNF-α, fecal calprotectin, and ESR before and after treatment (Table 5).

#### 2.5.1. Tumor Necrosis Factor-Alpha (TNF-α)

In the mesalamine group, the median TNF-α level significantly decreased from 303.5 pg/mL (IQR: 177.5–348.3) to 168 pg/mL (IQR: 155.8–298.3) (*p* = 0.003; Wilcoxon signed-rank test). In the PTX group, TNF-α showed a more pronounced reduction, from 340 pg/mL (IQR: 232.5–362.3) to 148 pg/mL (IQR: 124–262.5) (*p* < 0.001; Wilcoxon test). The between-group comparison after treatment revealed significantly lower TNF-α levels in the PTX group (*p* = 0.012; Mann–Whitney U test).

#### 2.5.2. Fecal Calprotectin

Fecal calprotectin levels were also significantly reduced in both groups following treatment. The mesalamine group showed a decrease from 257.2 µg/g (IQR: 207.4–282.6) to 109.1 µg/g (IQR: 73.88–188.8) (*p* < 0.001; Wilcoxon test). The PTX group showed a greater reduction from 243 µg/g (IQR: 188.6–362.3) to 78 µg/g (IQR: 53.63–108.4) (*p* < 0.001; Wilcoxon test). The post-treatment difference between the groups was statistically significant, favoring the PTX group (*p* = 0.033; Mann–Whitney U test).

Fecal calprotectin levels below 100 µg/g were achieved in 18 patients (66.7%) in the PTX group compared with 14 patients (53.8%) in the placebo group, while more stringent normalization of fecal calprotectin to levels below 50 µg/g was observed in six patients (20%) receiving PTX versus only two patients (6%) in the placebo group.

#### 2.5.3. Erythrocyte Sedimentation Rate

The ESR significantly decreased in both groups. In the mesalamine group, mean ESR dropped from 23.20 ± 5.38 mm/h to 13.33 ± 3.46 mm/h (*p* < 0.001; paired *t*-test), while in the PTX group, it declined from 23.90 ± 4.51 mm/h to 11.57 ± 4.15 mm/h (*p* < 0.001; paired *t*-test). The between-group comparison after treatment showed a significantly lower ESR in the PTX group (*p* = 0.027; unpaired *t*-test).

### 2.6. Comparison of Clinical and Biochemical Outcomes Between Treatment Groups in Mesalamine-Naïve Patients

In mesalamine-naïve patients, both treatment groups demonstrated significant improvements in clinical and biochemical outcomes following therapy. In the mesalamine group, the median PMS decreased from 5 (IQR: 4–6) to 2 (IQR: 1–3) (*p* = 0.002), while in the PTX group, it decreased from 4 (IQR: 3–6) to 1 (IQR: 0–1.75) (*p* = 0.001). The reduction in PMS was significantly greater in the PTX group compared to the mesalamine group (*p* = 0.023) (Table 6).

Similarly, quality of life, as assessed by IBDQ-32, improved significantly in both groups, with a greater increase observed in the PTX group (*p* = 0.006). Inflammatory markers showed significant reductions in both groups, including TNF-α (*p* = 0.004 and *p* = 0.0003, respectively), fecal calprotectin (*p* = 0.002 and *p* = 0.001, respectively), and ESR (*p* < 0.001 for both). Between-group comparisons demonstrated significantly greater improvements in the PTX group for TNF-α (*p* = 0.036), fecal calprotectin (*p* = 0.042), and ESR (*p* = 0.025).

### 2.7. Comparison of Clinical and Biochemical Outcomes Between Treatment Groups in Mesalamine-Experienced Patients

In mesalamine-experienced patients, both treatment groups exhibited significant improvements in clinical and biochemical outcomes following therapy. In the mesalamine group, the median PMS decreased from 5 (IQR: 5–6) to 3 (IQR: 1–4) (*p* = 0.001), while in the PTX group, it decreased from 5 (IQR: 3–5.75) to 2 (IQR: 0.25–2) (*p* < 0.001). The reduction in PMS was significantly greater in the PTX group compared to the mesalamine group (*p* = 0.016) (Table 7).

Quality of life, as measured by IBDQ-32, improved significantly in both groups, with a significantly greater increase observed in the PTX group (*p* = 0.043). Similarly, inflammatory markers demonstrated significant reductions in both groups, including TNF-α (*p* = 0.004 and *p* < 0.001, respectively), fecal calprotectin (*p* < 0.001 for both), and ESR (*p* < 0.001 for both). Between-group comparisons showed significantly greater improvements in the PTX group for TNF-α (*p* = 0.009), fecal calprotectin (*p* = 0.026), and ESR (*p* = 0.016).

Using ANCOVA with inclusion of an interaction term, no significant interaction was observed between treatment group and prior mesalamine exposure (*p* = 0.742), indicating that the treatment effect was consistent across subgroups.

### 2.8. Predictors of Clinical and Inflammatory Outcomes in the Studied Groups

To evaluate the potential influence of baseline demographic and disease-related variables on treatment outcomes, multivariable linear regression analyses were performed separately within each treatment group, adjusting for age, sex, disease duration, smoking status, and disease extent (E1–E3) (Table 8 and Table 9).

In the mesalamine group, none of the assessed variables were identified as significant predictors of clinical or biochemical outcomes. Specifically, no significant associations were observed between these covariates and changes in Inflammatory Bowel Disease Questionnaire (IBDQ-32) scores, partial Mayo score (PMS), fecal calprotectin, tumor necrosis factor-alpha (TNF-α), or erythrocyte sedimentation rate (ESR) (all *p* > 0.05).

Similarly, in the pentoxifylline (PTX) group, multivariable regression analysis demonstrated that none of the evaluated covariates significantly predicted clinical or inflammatory outcomes, including IBDQ-32, PMS, fecal calprotectin, TNF-α, and ESR (all *p* > 0.05).

Overall, these findings indicate that the observed improvements in clinical and inflammatory parameters were not significantly influenced by baseline demographic characteristics or disease-related factors, supporting the independence of the treatment effect from these potential confounders.

### 2.9. Correlation Analysis Between the Measured Variables

In the mesalamine group, a significant negative correlation was observed between the PMS and the IBDQ-32 score (r = −0.75, *p* < 0.001), and between IBDQ-32 scores and fecal calprotectin levels (r = −0.53, *p* = 0.001). Additionally, PMS showed a significant positive correlation with serum TNF-α levels (r = 0.517, *p* = 0.001) and with fecal calprotectin levels (r = 0.612, *p* < 0.001).

In the PTX group, the correlation between PMS and IBDQ-32 was similarly significant and negative (r = −0.633, *p* < 0.001), reflecting improved quality of life with reduced clinical disease activity. Moreover, IBDQ-32 scores showed a significant negative correlation with fecal calprotectin levels (r = −0.478, *p* = 0.001). The PTX group also demonstrated a significant positive correlation between PMS and fecal calprotectin levels (r = 0.702, *p* < 0.001) and with serum TNF-α levels (r = 0.451, *p* = 0.001).

### 2.10. Unadjusted and BH-FDR Adjusted p-Values for IBDQ Subdomains and Inflammatory Markers

To account for multiple comparisons across IBDQ subdomains and inflammatory markers, Benjamini–Hochberg false discovery rate (BH-FDR)-adjusted *p*-values were calculated. As shown in Table 10, the unadjusted *p*-values indicated statistically significant treatment effects of PTX on systemic domain (*p* = 0.0006), calprotectin (*p* = 0.003), TNF-α (*p* = 0.012), social domain (*p* = 0.015), ESR (*p* = 0.017), total IBDQ-32 score (*p* = 0.036), and digestive domain (*p* = 0.036). After BH-FDR adjustment, the results remained significant for systemic domain (*p* = 0.004), calprotectin (*p* = 0.012), TNF-α (*p* = 0.024), social domain (*p* = 0.030), ESR (*p* = 0.034), total IBDQ-32 (*p* = 0.048), and digestive domain (*p* = 0.048), confirming the robustness of the findings. The emotional domain was not statistically significant in either the unadjusted (*p* = 0.127) or adjusted analysis (*p* = 0.127).

### 2.11. Drug Safety and Tolerability

Drug-related side effects were monitored in both treatment groups and analyzed using Fisher’s exact test, as shown in Table 11. Vomiting was reported in 4 out of 26 patients (15.38%) in the mesalamine group and in 3 out of 27 patients (11.11%) in the PTX group (*p* = 0.704). Skin rash occurred in five patients (19.2%) receiving mesalamine and in four patients (14.8%) in the PTX group (*p* = 0.727). Flushing was reported by three mesalamine-treated patients (11.5%) compared to five patients (18.5%) in the PTX group (*p* = 0.704). Heartburn was experienced by two patients (7%) in the mesalamine group and four patients (14.8%) in the PTX group (*p* = 0.668). None of the differences in adverse events between the groups reached statistical significance. Overall, the incidence of side effects was comparable between the two groups, indicating that both mesalamine and PTX were well tolerated.

## 3. Discussion

To our knowledge, this study represents one of the first pilot clinical investigations to assess the adjunctive role of PTX in UC, integrating both clinical efficacy endpoints (PMS and IBDQ-32) and mechanistic inflammatory biomarkers such as fecal calprotectin, ESR, and TNF-α. The present study demonstrated that PTX, when used as an adjunctive therapy in patients with UC, significantly improved multiple clinical outcomes compared with placebo. Specifically, patients receiving PTX exhibited a greater reduction in the PMS, higher rates of clinical response and remission, and a marked improvement in IBDQ scores, reflecting better health-related quality of life.

In our study, the PTX group showed a substantial reduction in PMS from baseline to week 24, with median reductions exceeding those observed in the placebo group. This finding suggests that PTX may exert an anti-inflammatory effect sufficient to translate into measurable symptom relief. The reduction in PMS is consistent with PTX’s known ability to modulate inflammatory pathways, which are central to the pathogenesis of UC [10,20]. Clinical response and remission in PMS were achieved by a significantly greater proportion of patients in the PTX group than in the placebo group. These findings align with previous reports from preclinical studies, where PTX markedly reduced histopathological inflammation in colitis-induced animal models [21,22]. Clinical activity after adjustment improved in both treatment arms. However, the magnitude of improvement was more pronounced in the PTX group, and the between-group HL confidence interval indicated a meaningful treatment advantage. This finding is consistent with the observed biochemical improvements and offers clinical validation of pentoxifylline’s therapeutic impact. Improvements in PMS reinforce the premise that biochemical modulation translates into tangible clinical benefit for patients.

Beyond symptomatic control, PTX significantly improved IBDQ-32 scores compared with placebo. This improvement reflects better physical well-being, emotional status, and social functioning, which are critical endpoints in chronic disease management. Our results are consistent with the therapeutic profile of PTX, which acts as a non-selective phosphodiesterase inhibitor, increasing intracellular cyclic adenosine monophosphate (cAMP) levels and subsequently reducing the production of pro-inflammatory cytokines, such as TNF-α, IL-1β, and IL-6 [23,24]. Since these cytokines are key mediators of mucosal inflammation and systemic manifestations in UC, their suppression could directly translate into improvements in both physical and psychosocial domains of quality of life. The clinical significance of this improvement is underscored by the fact that even moderate increases in IBDQ-32 scores have been shown to correlate with higher rates of patient satisfaction and treatment adherence in IBD populations [25]. Although the between-group HL confidence interval was narrower than that for the biochemical markers, the statistically significant difference suggests that patients derive real-life benefit beyond laboratory and clinical scores. Given that UC has substantial psychosocial and functional implications, this improvement in quality of life is clinically important. Although data on PTX in UC are scarce, similar benefits in health-related quality of life have been documented in other chronic inflammatory conditions, such as Crohn’s disease [26], and rheumatoid arthritis [27], where PTX reduced systemic inflammation and improved functional status.

In our study, PTX administration resulted in a significant reduction in serum TNF-α levels compared to the placebo group. Elevated TNF-α levels correlate with disease activity and mucosal injury, and their reduction is associated with clinical remission and mucosal healing [28]. The observed TNF-α suppression with PTX is consistent with its known pharmacological action as a phosphodiesterase inhibitor, which increases intracellular cAMP and subsequently downregulates TNF-α gene transcription [10,29]. These findings align with preclinical studies in experimental colitis models, where PTX administration markedly reduced TNF-α concentrations and ameliorated histopathological inflammation [13,15]. This finding is clinically relevant, given the central role of TNF-α in mediating mucosal inflammation and its established association with disease severity and relapse risk in UC.

Erythrocyte sedimentation rate (ESR) also decreased significantly in the PTX group compared to placebo. These results were in line with previous reports [30,31]. Although ESR is influenced by multiple factors, it remains a valuable indicator of inflammatory burden in IBD and correlates with clinical disease activity [32,33]. The reduction in ESR observed here supports the systemic anti-inflammatory effects of PTX beyond localized mucosal modulation [34]. Although ESR improved significantly, we acknowledge its limited current clinical utility and have retained it primarily for completeness.

Fecal calprotectin showed a substantial decline in patients receiving PTX compared with placebo. The attenuation of fecal calprotectin suggests that PTX effectively reduces neutrophil-driven mucosal inflammation, potentially contributing to improved mucosal healing rates. This is in agreement with prior animal studies where PTX treatment mitigated neutrophil infiltration into colonic tissue [35]. From a clinical perspective, different thresholds of fecal calprotectin have distinct implications. Levels below 100 µg/g are generally considered indicative of reduced inflammatory activity and are associated with a lower risk of relapse, whereas normalization below 50 µg/g is more closely linked to mucosal healing and deep remission. In the present study, a higher proportion of patients in the PTX group achieved calprotectin levels below 100 µg/g compared with the placebo group, suggesting improved inflammatory control. However, the proportion of patients achieving more stringent normalization below 50 µg/g remained relatively low, indicating that while PTX may contribute to partial biochemical remission, its effect on achieving deep mucosal healing may be limited. These findings highlight the need for cautious interpretation of biomarker improvements and reinforce the importance of more stringent therapeutic targets in future studies.

The clinical relevance of the observed reductions in inflammatory biomarkers warrants careful consideration. In this study, the significant decreases in TNF-α, fecal calprotectin, and ESR in the PTX group were paralleled by improvements in clinical outcomes, including reduced disease activity and enhanced quality of life, suggesting that these biomarker changes are not merely biochemical findings but reflect meaningful therapeutic effects. In particular, fecal calprotectin is a well-established surrogate marker of intestinal inflammation and correlates with mucosal healing; thus, its substantial reduction supports the potential of PTX to promote deeper disease control. Moreover, reductions in TNF-α, a central pro-inflammatory cytokine in ulcerative colitis pathogenesis, further reinforce the biological plausibility of the observed clinical benefits. While complete normalization of biomarkers was not consistently achieved in all patients, the observed shifts toward lower inflammatory burden are clinically relevant, as even partial reductions have been associated with decreased relapse risk and improved long-term outcomes.

Taken together, the parallel reduction in TNF-α, ESR, and fecal calprotectin highlights PTX’s multi-targeted anti-inflammatory potential in UC. The convergence of these biomarker improvements with clinical benefits observed in our trial suggests that PTX may represent a cost-effective adjunctive therapy in UC, particularly in resource-limited settings or in patients who are not candidates for biologics. Several randomized clinical trials investigating other therapeutic agents in IBD have also assessed inflammatory biomarkers similar to those evaluated in the present study. For example, a randomized double-blind placebo-controlled trial evaluating curcumin with or without piperine in patients with IBD measured fecal calprotectin, pro-inflammatory cytokines, and oxidative stress markers before and after treatment, demonstrating modulation of inflammatory pathways following intervention [36]. Likewise, trials evaluating biologic therapies such as mirikizumab demonstrated that decreases in fecal calprotectin and CRP were strongly associated with histologic and endoscopic improvement [37]. In line with the randomized controlled study by Bahaa et al. (2024), which demonstrated that PTX modulates key inflammatory pathways including IL-6/STAT3, zonula occludin 1 (ZO-1), and sphingosine 1 phosphate (S1P), our trial observed significant reductions in TNF-α, fecal calprotectin, and ESR, accompanied by improvements in clinical disease activity and quality of life [10]. Moreover, case reports such as that by Jiménez-Luévano et al. (2024) have illustrated the potential of PTX to ameliorate extraintestinal manifestations of UC, including pyoderma gangrenosum, supporting the broader anti-inflammatory effects of PTX [17]. These findings support the use of inflammatory biomarkers as reliable indicators of therapeutic response in UC and provide a useful framework for interpreting the biomarker improvements observed with adjunctive PTX therapy in the present study. While the results were statistically significant, these improvements are clinically modest and should be interpreted in that context.

Pentoxifylline exerts multiple mechanistic effects that may be relevant to UC, including well-established anti-inflammatory and immunomodulatory actions, as well as hemorheological properties that improve blood flow [38]. Its anti-inflammatory activity—mediated through inhibition of TNF-α synthesis, suppression of NF-κB signaling, reduction in oxidative stress, and attenuation of leukocyte activation [39]—appears to be the mechanism most directly aligned with the observed reductions in TNF-α, fecal calprotectin, and ESR. In contrast, its hemorheological effects, such as decreased blood viscosity, enhanced erythrocyte deformability, and improved capillary perfusion, may offer supportive microcirculatory benefits that facilitate mucosal repair [40]. However, these effects alone are unlikely to account for the magnitude and consistency of the biochemical improvements observed. Overall, the findings of this study primarily reflect the anti-inflammatory actions of pentoxifylline, with its rheological properties likely contributing as ancillary supportive mechanisms.

The multivariable regression analyses conducted within both treatment groups showed that demographic and disease-related characteristics—including age, sex, disease duration, smoking status, and disease extent—were not significant predictors of clinical or biochemical outcomes. This pattern was consistent across key endpoints such as PMS, fecal calprotectin, TNF-α, and ESR, indicating that baseline patient factors did not materially influence treatment response. In the mesalamine group, the absence of significant predictors suggests a uniform response pattern, while the PTX group similarly showed no covariates exerting a measurable impact on outcomes. Clinically, this implies that the benefits observed with adjunctive PTX are likely consistent across different patient subgroups, strengthening the internal validity and robustness of the treatment effect. These results are in line with previous literature, indicating that key inflammatory pathways in UC, particularly those involving pro-inflammatory cytokines such as TNF-α, tend to operate independently of demographic variables, and that treatment response is more closely related to modulation of these biological pathways than to baseline patient characteristics [41,42,43]. Clinical trial data indicate that age of disease onset and duration do not meaningfully modify treatment response when adjusted for severity and other variables, supporting the robustness of our findings [43]. Additionally, meta-analytic data suggest that sex alone does not substantially alter key clinical outcomes in UC, further reinforcing that treatment effects may largely operate independently of baseline demographic factors [44].

To further address potential heterogeneity related to prior treatment exposure, subgroup analyses were conducted stratifying patients into mesalamine-naïve and mesalamine-experienced groups. Notably, the beneficial effects of PTX were consistently observed across both subgroups, with significant improvements in disease activity, inflammatory markers, and quality of life. Importantly, the magnitude and direction of treatment effects were comparable between the overall analysis and the subgroup analyses, indicating that prior mesalamine exposure did not materially influence the response to adjunctive PTX therapy.

These findings suggest that PTX may exert its therapeutic effects independently of baseline treatment status, supporting its role as a promising adjunctive therapy in patients with mild-to-moderate ulcerative colitis. Moreover, the consistency of results across the total population and stratified analyses strengthens the internal validity of the study and mitigates concerns regarding population heterogeneity. However, given the relatively small sample size within subgroups, these findings should be interpreted with caution and warrant confirmation in larger, adequately powered trials.

The correlation analyses demonstrated consistent relationships between clinical disease activity, inflammatory biomarkers, and quality of life across both groups. Higher PMS was associated with increased TNF-α and fecal calprotectin levels and lower IBDQ-32 scores, while higher inflammatory markers were linked to poorer quality of life. These findings reflect a coherent pattern in which greater inflammatory burden corresponds to worse clinical status and patient-reported outcomes, with similar trends observed in both treatment groups. The correlation analyses in this study should be interpreted as descriptive associations rather than evidence of causal relationships. Although these associations may be influenced by multiple confounding factors, their consistency across treatment groups and their alignment with established disease mechanisms support the internal validity and biological plausibility of the findings.

In this study, both ITT and PP analyses were performed to assess the efficacy of adjunctive PTX in patients with UC. Despite these methodological differences, the results of both approaches were largely consistent. Significant improvements in PMS, clinical response and remission rates, inflammatory markers (TNF-α, fecal calprotectin, and ESR), and disease-specific quality of life (IBDQ-32) were observed in the PTX group compared to mesalamine alone in both ITT and PP analyses. Numerical differences were minimal, reflecting the influence of excluded patients in the PP set, but the direction and magnitude of treatment effects remained concordant. This consistency reinforces the robustness of the observed treatment benefits and suggests that the study findings are not substantially biased by the analytical approach. Future studies may consider more advanced imputation methods, such as multiple imputation, to further validate these findings.

To account for the risk of type I error arising from multiple secondary outcomes, including IBDQ subdomains and inflammatory markers (TNF-α, calprotectin, and ESR), we applied the Benjamini–Hochberg false discovery rate (BH-FDR) procedure. Both unadjusted and BH-FDR-adjusted *p*-values are reported. After adjustment, all primary findings—including improvements in total IBDQ score, TNF-α, ESR, calprotectin, and key IBDQ subdomains (social, systemic, and digestive)—remained statistically significant, confirming the robustness of the observed treatment effects. The emotional domain, originally non-significant, remained non-significant after adjustment. This approach ensured control of the false discovery rate while preserving statistical power, providing a more reliable interpretation of multiple correlated outcomes without over-conservatism associated with methods such as Bonferroni correction.

Although the sample size was determined based on pilot study considerations, effect size estimation revealed a moderate-to-large treatment effect. This magnitude suggests that the observed differences are likely to be clinically meaningful despite the limited sample size. Furthermore, the consistency of effect sizes across both intention-to-treat and per-protocol analyses reinforces the robustness and reliability of the observed treatment effect, while supporting the exploratory nature of the study and the need for larger, adequately powered confirmatory trials.

In the present study, PTX was generally well tolerated, with no statistically significant differences in the incidence or severity of adverse events between the PTX and placebo groups. The reported side effects were mostly mild and none required discontinuation of therapy. These findings are consistent with previous clinical conditions [45,46,47], including inflammatory bowel disease [10,26].

This trial has several methodological and clinical strengths that increase confidence in the findings. It used a randomized, double-blind, placebo-controlled design, which minimizes selection, performance, and detection bias. The study evaluated a broad range of clinically relevant endpoints—including the PMS and the IBDQ-32—together with objective inflammatory biomarkers (serum TNF-α, fecal calprotectin, and ESR), allowing concordant assessment of symptom, biochemical, and patient-centered outcomes. We applied an intention-to-treat (ITT) analytic approach and handled missing data using a conservative baseline-observation-carried-forward method, thereby preserving randomization and limiting attrition bias.

Several limitations should be considered when interpreting the findings. First, the 24-week duration limits the assessment of long-term outcomes, including durability of response, maintenance of remission, and long-term safety of PTX. Second, the absence of mandatory endoscopic follow-up precluded direct evaluation of mucosal healing, a key therapeutic target in UC. Although the use of the PMS was justified by practical and ethical considerations, it limits comprehensive endoscopic assessment; therefore, the observed improvements should not be interpreted as definitive evidence of mucosal healing. Nonetheless, the consistent reductions in objective inflammatory biomarkers, particularly fecal calprotectin and TNF-α, provide supportive evidence of decreased intestinal inflammation. Clinical remission was defined using the PMS, and endoscopic assessment was not performed. Therefore, the reported remission rates reflect clinical and biochemical improvements but may not fully capture mucosal healing, which should be considered in interpreting the results.

This study was conducted at a single center with a relatively homogeneous patient population, which may limit the generalizability of the findings to broader and more diverse clinical settings. Therefore, the results should be interpreted with caution. Furthermore, the success of blinding was not formally assessed, which introduces a potential risk of bias. Finally, although patients received standardized counseling, unmeasured factors such as dietary variations cannot be entirely excluded. Despite these limitations, the consistency of findings across multiple clinical and biochemical outcomes, as well as across subgroup and adjusted analyses, supports the robustness of the observed treatment effect. Larger, multicenter trials with longer follow-up and endoscopic endpoints are warranted to confirm these results.

## 4. Materials and Methods

Between February 2023 and May 2025, sixty eligible patients were enrolled from the Internal Medicine Department, Faculty of Medicine, Fayoum University. The study was approved by the Institutional Review Board (Approval No. M 416, date of approval 17 March 2023) and conducted in accordance with the Helsinki Declaration. Participation was voluntary, and patients could withdraw at any time. Both patients and physicians were blinded to treatment allocation.

### 4.1. Inclusion Criteria

Eligible patients were adults (≥18 years) of either sex with mild-to-moderate UC. Mild-to-moderate UC was defined as a partial Mayo score (PMS) between 3 and 6, with no individual sub-score exceeding 2, and without systemic toxicity [48]. Both mesalamine-naïve patients and those already receiving mesalamine therapy were included.

### 4.2. Exclusion Criteria

The exclusion criteria included patients with colorectal cancer, severe UC, or those who were breastfeeding; individuals with significant liver or kidney function abnormalities; patients receiving rectal or systemic steroids, antiplatelet or anticoagulant therapy, and/or immunosuppressive or biological treatments; and those with a known allergy to the studied medications. Severe UC was defined as PMS > 6 or the presence of systemic toxicity (fever, tachycardia, and anemia).

### 4.3. Study Design

**Figure 1:**
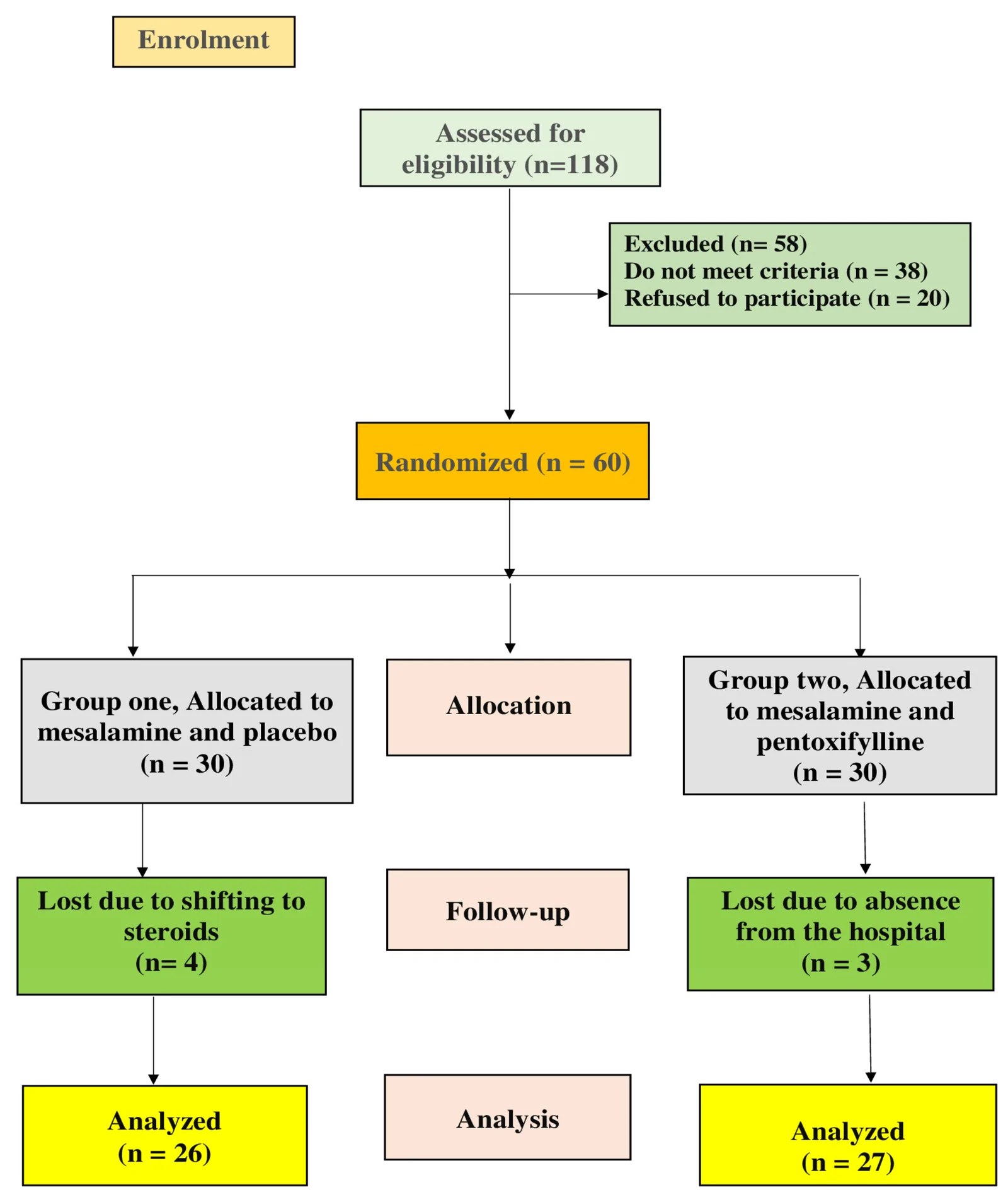
CONSORT diagram showing the flow of participants during the study.

This study was conducted as a prospective, pilot, randomized, double-blind clinical trial and was prospectively registered on ClinicalTrials.gov (NCT05575505) on 20 February 2023. A total of 60 eligible patients with ulcerative colitis were enrolled and randomly allocated into two treatment arms, as illustrated in the CONSORT flow diagram (Figure 1). Randomization was executed using a computer-generated block randomization sequence with variable block sizes (4 and 6) to ensure balanced distribution between groups. To maintain allocation concealment, sequentially numbered, opaque, sealed envelopes were prepared by an independent staff member who was not involved in participant recruitment, clinical management, or outcome evaluation. Blinding was upheld throughout the study for both participants and treating physicians. Written informed consent was obtained from all participants prior to their inclusion in the trial.

Group 1 (Placebo Group): Participants in this arm were administered an inert placebo tablet twice per day, in combination with mesalamine at a total daily dose of 3 g, given as 1 g three times daily (Pentasa^®^ 500 mg, Multi Pharma, Alexandria, Egypt), for a treatment duration of six months.

Group 2 (PTX Group): Individuals assigned to this group received PTX at a dose of 400 mg twice daily (Trental^®^ 400 mg, Sanofi, Cairo, Egypt), together with mesalamine 1 g taken three times daily, for a total of six months. The placebo tablets were identical in appearance to the PTX tablets.

### 4.4. Sample Size Calculations

At the time of planning this study, no published data were available regarding the effect size of PTX on PMS in patients with UC. Therefore, this trial was designed as a pilot study, following the recommendations of Sim and Lewis [49] who suggested that a sample size of more than 22 participants per group is appropriate for detecting a small-to-medium effect size while minimizing combined Type I and Type II errors. Anticipating a potential dropout rate of 20%, the target sample size was increased to 30 participants per group. Participants were randomly assigned to each group, with the α-error set at 0.05 (two-tailed). Given the exploratory nature of the study, a formal a priori power calculation based on a predefined effect size was not performed. Instead, the primary objective was to generate preliminary data on the efficacy and safety of PTX as an adjunctive therapy in UC and to inform the design and sample size estimation of future adequately powered randomized controlled trials.

### 4.5. Study Protocol and Follow-Up

Participants were closely monitored over the 6-month study duration to evaluate treatment adherence, disease trajectory, and the emergence of any adverse events. At enrollment, all individuals underwent a comprehensive baseline assessment that included a detailed medical history, physical examination, and a full panel of laboratory investigations to exclude underlying systemic or organ-specific dysfunction. Baseline laboratory workup consisted of complete blood count, hepatic and renal function tests, metabolic screening, and key inflammatory biomarkers—serum TNF-α, ESR, and fecal calprotectin—which served as objective indicators of intestinal inflammation. All laboratory measurements were performed in the same accredited facility to ensure analytical consistency, and cytokine concentrations were quantified using validated ELISA kits following standardized manufacturer protocols. Throughout the study, participants attended monthly in-person clinic visits for clinical evaluation, disease activity scoring, repeat laboratory testing, and assessment of medication adherence, which was monitored through pill counts and patient reporting. Adherence assessment was further supported by direct patient interviews during these visits and reinforced through weekly telephone follow-ups, during which compliance, missed doses, new symptoms, and potential adverse events were systematically assessed. Hospital records were reviewed regularly throughout the study to identify any adverse events or hospital admissions that were not reported during scheduled visits. Study medications were dispensed monthly in pre-labeled containers, and placebo tablets were manufactured to be indistinguishable from PTX in appearance, packaging, and administration schedule to ensure maintenance of blinding. Both participants and treating clinicians remained unaware of treatment allocation for the duration of the trial. Adverse events were actively monitored through direct patient questioning during scheduled clinic visits and weekly telephone follow-ups; however, a formal structured questionnaire for systematic adverse event collection was not employed.

All participants received standardized nutritional and lifestyle counseling at baseline and were advised to avoid major dietary changes during the study. Participants were also advised to avoid the use of over-the-counter supplements with potential anti-inflammatory effects, and were routinely questioned during follow-up visits and telephone contacts regarding any new supplement use or significant dietary changes. Concomitant use of NSAIDs, corticosteroids, immunosuppressants, or biologic therapies was prohibited throughout the trial.

The selected mesalamine dose (1 g t.i.d.) was based on established treatment guidelines for mild-to-moderate UC [50] while the PTX dose (400 mg Bid) was chosen based on prior clinical and preclinical studies demonstrating anti-inflammatory efficacy [45]. The selected dose of PTX (400 mg twice daily) was based on its established clinical use and favorable safety profile. This dosing regimen is widely used in clinical practice and has demonstrated anti-inflammatory effects, particularly through inhibition of TNF-α production [10,51]. Bahaa et al. reported that PTX 400 mg BID achieved anti-inflammatory effects in patients with mild-to-moderate UC [10].

### 4.6. Study Outcomes

Given that the study population included both mesalamine-naïve and previously treated patients, subgroup analyses were planned to evaluate whether prior mesalamine exposure influenced the response to adjunctive PTX. Outcomes were compared between mesalamine-naïve and experienced participants for the primary and secondary endpoints to assess potential heterogeneity in treatment effect.

#### 4.6.1. Primary Outcomes

The study’s primary endpoint was the change in the partial Mayo score (PMS), a measure used to evaluate remission and clinical response in patients with mild-to-moderate UC. Although the trial was initially registered to use the complete Mayo score, disease activity during the study was assessed using the PMS due to practical and ethical constraints related to repeated endoscopic evaluation in patients with mild-to-moderate UC, particularly in the absence of clinical deterioration; this modification was applied uniformly across both study groups and is unlikely to affect the validity of the findings given that the PMS is a validated and widely accepted measure of clinical disease activity. The PMS is a validated and widely accepted measure of clinical disease activity in UC and has been extensively used in clinical trials and real-world studies. It has demonstrated a strong correlation with the full Mayo score and endoscopic findings, supporting its use as a reliable surrogate endpoint when repeated endoscopic assessment is not feasible or ethically justified. The PMS, which excludes the endoscopic sub-score, is a validated and widely used clinical tool for evaluating disease activity, response, and remission in UC and was applied uniformly across both treatment groups.

#### 4.6.2. Secondary Outcomes

Secondary endpoints included changes in the Inflammatory Bowel Disease Questionnaire (IBDQ-32) scores, along with alterations in serum TNF-α, ESR, and fecal calprotectin concentrations, collectively providing a comprehensive assessment of the physiological and inflammatory responses associated with PTX therapy.

### 4.7. Evaluation of Colitis

The partial Mayo score (PMS) was utilized to evaluate disease activity in patients with UC. This non-invasive index comprises three components: stool frequency, rectal bleeding, and the physician’s global assessment, yielding a total score between 0 and 9 [48]. PMS measurements were obtained at baseline and again at the end of the study period. Clinical response was defined as either a reduction of ≥1 point in the rectal bleeding subscore or an absolute rectal bleeding subscore of 0 or 1, together with a decrease in the total PMS of ≥2 points and ≥30% from baseline. Clinical remission was defined as a PMS of less than 2, with no individual subscore exceeding 1 [52]. A 24-week follow-up period is consistent with previous UC studies, such as the GO-COLITIS trial, which used a similar timeframe to evaluate induction and maintenance therapy outcomes [25]. This duration provides sufficient time to observe treatment-related effects while ensuring patient adherence and safety monitoring.

### 4.8. Assessment of Quality of Life

The IBDQ-32 is the most widely used tool for evaluating disease-specific quality of life in randomized clinical trials involving UC. It measures four domains: emotional function, social function, bowel symptoms, and systemic symptoms [53]. Evidence from reviews of its measurement properties confirms its responsiveness, construct validity, reliability, and content validity [54,55]. The total score, ranging from 32 to 224, is obtained by summing the scores of all 32 items, with higher scores indicating better health-related quality of life.

### 4.9. Sample Collection

Before the study commenced and once more at six months following the intervention, a 10 mL sample of venous blood was collected from the antecubital vein. The specimens were carefully placed into collection tubes, left to clot at room temperature, and subsequently centrifuged at 4500× *g* for 10 min using a Hettich Zentrifugen EBA 20 device. The separated serum was portioned into two fractions: one was preserved at −80 °C for later determination of cytokine concentrations, while the other was utilized immediately for routine evaluations of liver and kidney function parameters.

### 4.10. Biochemical Analysis

Serum TNF-α (catalog no. 201-12-0083) and fecal calprotectin (catalog no. 201-12-5461) were quantified using commercially available ELISA kits (SunRed, Shanghai, China), following the manufacturer’s standardized protocols.

### 4.11. Handling of Missing Data

Missing outcome data resulting from loss to follow-up were handled using an intention-to-treat (ITT) approach to preserve the benefits of randomization and minimize potential bias. Missing outcome data were addressed using the baseline-observation-carried-forward (BOCF) approach, a highly conservative method that assumes no change from baseline in participants with missing follow-up data. While this approach may bias the results toward the null and potentially underestimate treatment effects, it was chosen to preserve the integrity of randomization and minimize inflation of efficacy estimates in this pilot study. We acknowledge that alternative imputation methods (e.g., multiple imputation or last observation carried forward) might yield different estimates, and the limitations of BOCF should be considered when interpreting the findings.

Patients who required escalation of therapy due to clinical deterioration (e.g., initiation of corticosteroids) were considered treatment failures during follow-up and were retained in the ITT analysis, with missing outcome data handled using the BOCF method. Although this is a highly conservative approach that may bias the results toward the null, it was selected to maintain the integrity of randomization and to prevent overestimation of treatment effects in this pilot study. In addition to the ITT analysis using the conservative BOCF method, we have now conducted a per-protocol (PP) analysis for the primary outcome (partial Mayo score), including only participants who completed the study without major protocol deviations. This complementary analysis supports the robustness of our findings.

### 4.12. Statistical Analysis

Data analysis was carried out using Prism software, version 9 (GraphPad Software, Inc., San Diego, CA, USA). The distribution of continuous variables was assessed using multiple approaches, including the Shapiro–Wilk test, skewness and kurtosis indices, and visual inspection of histograms and Q-Q plots. For comparisons within each group, non-parametric variables were analyzed using the Wilcoxon signed-rank test, whereas parametric variables were assessed with the paired Student’s *t*-test, comparing pre- and post-treatment values. Differences between the two groups, both before and after the intervention, were analyzed using the Mann–Whitney U test for non-parametric data and the unpaired Student’s *t*-test for parametric data.

Categorical variables were summarized as counts and percentages, while continuous variables were expressed either as medians with interquartile ranges (IQR) or as means ± standard deviation (SD), depending on the distribution. Associations between categorical variables were evaluated using either Fisher’s exact test or the Chi-square test. Relationships between variables that did not follow a normal distribution were explored using Spearman’s rank correlation. Additionally, multilinear regression analysis was performed to assess whether age, sex, smoking status, disease duration, and disease extent influenced the primary and secondary study outcomes. An analysis of covariance (ANCOVA) model including an interaction term between treatment and prior mesalamine exposure was used to assess effect modification. To account for multiple comparisons across secondary outcomes, the Benjamini–Hochberg false discovery rate (BH-FDR) procedure was applied. Both unadjusted and BH-FDR-adjusted *p*-values are reported. All statistical tests were two-sided, and a *p*-value of less than 0.05 was considered indicative of statistical significance.

## 5. Conclusions

This pilot randomized trial provides preliminary evidence that adjunctive PTX may improve clinical and biochemical outcomes in patients with mild-to-moderate UC. However, due to the exploratory design and limited sample size, these findings should be interpreted cautiously and require confirmation in larger, adequately powered studies. Given its established safety profile, PTX may represent a feasible repurposed adjunct rather than a replacement for existing treatments. These findings should be interpreted in light of the study’s limitations, including the single-center design and relatively short follow-up period. Further large-scale, multicenter studies with longer follow-up are needed to confirm these results and to better define the clinical positioning of PTX in UC management.

## Figures and Tables

**Table 1:** Clinical, demographic and laboratory data of the patients.

Parameter	Mesalamine Group (n = 30)	PTX Group (n = 30)	*p*-Value
Age (years)	41.80 ± 8.48	39.27 ± 11.25	0.329
Sex (M/F)	14/16	17/13	0.438
Weight (kg)	67.27 ± 6.08	69.87 ± 6.16	0.105
BMI (kg/m^2^)	22.62 ± 1.51	22.86 ± 2.08	0.599
Serum ALT (IU/L)	28.83 ± 5.29	29.37 ± 3.102	0.636
Serum AST (IU/L)	31.90 ± 4.98	33.13 ± 6.86	0.421
SrCr (mg/dL)	0.94 ± 0.13	0.95 ± 0.10	0.678
Hgb (mg/dL)	13.06 ± 1.07	12.74 ± 1.10	0.253
Albumin (g/dL)	4.35 (3.4–5.3)	3.85 (3.49–5.6)	0.950
Platelet (mcL × 10^3^)	195.7 ± 16.71	200.9 ± 13.43	0.187
Disease duration	0.7 (0–2.45)	0.5 (0–1.72)	0.590
Naïve (no.)	11	14	0.614
Experience disease (no.)	19	16	0.686
Smoking (no.)	7	5	0.518
Site of disease (no.)			
Proctitis	14	10	0.518
Left-sided	10	11	
Extensive colitis	6	9	

Data are presented as mean ± SD, median, interquartile range, and numbers. Mesalamine group, UC patients treated with mesalamine and placebo; PTX group, UC patients treated with mesalamine plus pentoxifylline; M, Male; F, Female; ALT, alanine aminotransferase; AST, aspartate aminotransferase; Hgb, hemoglobin; Sr Cr, serum creatinine. Significance at (*p* < 0.05).

**Table 2:** Effect of study medications on clinical markers using intention-to-treat analysis.

Character	Placebo Group (n = 30)			PTX Group (n = 30)			*p*-Value
	Before Treatment	After Treatment	*p*-Value	Before Treatment	After Treatment	*p*-Value	After Treatment
Partial Mayo score index (PMS)	5 (5–6)	2 (1–3)	<0.001 *	5 (3–6)	1 (0–2)	<0.001 *	0.004 **
% change	60%			80%			
Response (n, %)	15 (50%)			23 (76.66%)			0.032 ***
Remission (n, %)	7 (23.33%)			15 (50%)			0.04 ***

Data are presented as numbers, median, percentages, and interquartile range. Mesalamine group, UC patients treated with mesalamine and placebo; PTX group, UC patients treated with mesalamine plus pentoxifylline. (*) level of significance within the same group using Wilcoxon test. (**) level of significance between groups using Mann–Whitney U test. (***) level of significance between groups using Chi-square test. Significance at (*p* < 0.05).

**Table 3:** Effect of study medications on clinical markers by per-protocol analysis.

Character	Placebo Group (n = 26)			PTX Group (n = 27)			*p*-Value
	Before Treatment	After Treatment	*p*-Value	Before Treatment	After Treatment	*p*-Value	After Treatment
PMS	5 (4.5–6)	2 (1–3)	<0.0001 *	5 (3–6)	0.75 (0–2)	<0.0001 *	0.0006 **
Response (n, %)	15 (57.7%)			23 (85.18%)			0.02 ***
Remission (n, %)	7 (26.9%)			15 (55.55%)			0.034 ***

Data are presented as numbers, median, percentages, and interquartile range. Mesalamine group, UC patients treated with mesalamine and placebo; PTX group, UC patients treated with mesalamine plus pentoxifylline; PMS, partial Mayo score. (*) level of significance within the same group using Wilcoxon test. (**) level of significance between groups using Mann–Whitney U test. (***) level of significance between groups using Chi-square test. Significance at (*p* < 0.05).

**Table 4:** Effect of study medications on disease-specific quality of life via IBDQ subscales.

Character	Group 1 Mesalamine Group (n = 30)			% Change Within Group	Group 2 PTX Group (n = 30)			^##^ *p*-Value	% Change Within Group
	Before Treatment	After Treatment	^#^ *p*-Value		Before Treatment	After Treatment	^#^ *p*-Value	After Treatment	
Social domain	16 (11–21)	18.5 (13–24.5)	0.03	+15.6%	15 (11.75–20)	23 (19.75–28)	<0.001	0.015	+53.3%
Systemic domain	15.5 (9–19.25)	18.5 (15.75–21)	0.003	+19.4%	16.5 (10.75–22.25)	22.5 (19.75–27)	<0.001	0.006	+36.4%
Digestive domain	34 (31.75–37)	53.5 (36–59)	<0.001	+57.4%	34 (25–38)	57 (50.75–64)	<0.001	0.036	+67.6%
Emotional domain	22 (16.5–31)	31 (19–39)	0.017	+40.9%	27 (15.5–38.5)	36 (24.75–42.25)	<0.001	0.127	+33.3%
Total IBDQ score	85 (71–102)	121 (104.5–146.3)	<0.001	+42.4%	100 (74.75–109)	135.5 (119.3–149)	<0.001	0.036	+35.5%

Data are presented as median and interquartile range. Mesalamine group, UC patients treated with mesalamine and placebo; PTX group, UC patients treated with mesalamine plus pentoxifylline. (^#^) level of significance within group using Wilcoxon test. (^##^) level of significance between groups using Mann–Whitney U test. IBDQ, Inflammatory Bowel Disease Questionnaire. Significance at (*p* < 0.05).

**Table 5:** Effect of study medications on serum and fecal parameters.

	Mesalamine Group (n = 30)			% Change Within Group	PTX Group (n = 30)			% Change Within Group	*p*-Value
Character	Before Treatment	After Treatment	*p*-Value		Before Treatment	After Treatment	*p*-Value		After Treatment
TNF-α (pg/mL)	303.5 (177.5–348.3)	168 (155.8–298.3)	0.003 ^A^	−44.7%	340 (232.5–362.3)	148.0 (124–262.5)	<0.001 ^A^	−56.5%	0.012 ^C^
Calprotectin (µg/g)	257.2 (207.4–282.6)	109.1 (73.88–188.8)	<0.001 ^A^	−57.6%	243 (188.6–362.3)	78 (53.63–108.4)	<0.001 ^A^	−67.9%	0.033 ^C^
ESR (mm/h)	23.20 ± 5.38	13.33 ± 3.46	<0.001 ^B^	−42.5%	23.90 ± 4.51	11.57 ± 4.15	<0.001 ^B^	−51.6%	0.027 ^D^

Data are presented as mean ± SD, median, and interquartile range. Mesalamine group, UC patients treated with mesalamine and placebo; PTX group, UC patients treated with mesalamine plus pentoxifylline; TNF-α, tumor necrosis factor-alpha; ESR, erythrocyte sedimentation rate. (^A^ and ^B^) level of significance within the same group by Wilcoxon test and paired *t*-test, respectively. (^C^ and ^D^) level of significance between groups using Mann–Whitney U test and unpaired *t*-test, respectively. Significance at (*p* < 0.05).

**Table 6:** Comparison of clinical and biochemical outcomes between treatment groups in mesalamine-naïve patients.

Character	Mesalamine Group (n = 11)			PTX Group (n = 14)			*p*-Value After Treatment
	Before Treatment	After Treatment	*p*-Value	Before Treatment	After Treatment	*p*-Value	After Treatment
PMS	5 (4–6)	2 (1–3)	0.002 ^A^	4 (3–6)	1 (0–1.75)	0.001 ^A^	0.023 ^C^
IBDQ-32	85 (75–102)	120 (105–139)	0.001 ^A^	90.5 (74.57–112.5)	138.7 (114–173.5)	0.0001 ^A^	0.006 ^C^
TNF-α (pg/mL)	294.8 (187.2–331)	175.6 (145.5–236.8)	0.004 ^A^	284.8 (192.3–342.5)	157.4 (128.6–210.3)	0.0003 ^A^	0.036 ^C^
Calprotectin (µg/g)	239.4 (120.9–282.2)	84 (73–184)	0.002 ^A^	257.6 (204.4–355.3)	65.90 (47.15–129.7)	0.0001 ^A^	0.042 ^C^
ESR (mm/h)	20.18 ± 3.81	12.36 ± 3.23	<0.0001 ^B^	21.86 ± 3.95	10.79 ± 3.94	<0.0001 ^B^	0.025 ^D^

Data are presented as mean ± SD, median, and interquartile range. Mesalamine group, UC patients treated with mesalamine and placebo; PTX group, UC patients treated with mesalamine plus pentoxifylline; TNF-α, tumor necrosis factor-alpha; ESR, erythrocyte sedimentation rate. (^A^ and ^B^) level of significance within the same group by Wilcoxon test and paired *t*-test, respectively. (^C^ and ^D^) level of significance between groups using Mann–Whitney U test and unpaired *t*-test, respectively. Significance at (*p* < 0.05).

**Table 7:** Comparison of clinical and biochemical outcomes between treatment groups in mesalamine-experienced patients.

Character	Mesalamine Group (n = 16)			PTX Group (n = 19)			*p*-Value After Treatment
	Before Treatment	After Treatment	*p*-Value	Before Treatment	After Treatment	*p*-Value	After Treatment
PMS	5 (5–6)	3 (1–4)	0.001 ^A^	5 (3–5.75)	2 (0.25–2)	<0.001 ^A^	0.016 ^C^
IBDQ-32	85 (68–102)	126 (103–147)	<0.001 ^A^	89 (73.5–108.8)	140.5 (123–152.8)	<0.001 ^A^	0.043 ^C^
TNF-α (pg/mL)	328 (181–352)	184 (157–303)	0.004 ^A^	349.5 (232.5–375.5)	130 (121–267)	<0.001 ^A^	0.009 ^C^
Calprotectin (µg/g)	259.6 (209.2–283.5)	118 (78–193.2)	<0.001 ^A^	213.1 (139.1–367.3)	85 (68.13–110)	<0.001 ^A^	0.026 ^C^
ESR (mm/h)	24.95 ± 5.46	13.89 ± 3.55	<0.001 ^B^	25.69 ± 4.30	10.50 ± 4.36	<0.001 ^B^	0.016 ^D^

Data are presented as mean ± SD, median, and interquartile range. Mesalamine group, UC patients treated with mesalamine and placebo; PTX group, UC patients treated with mesalamine plus pentoxifylline; TNF-α, tumor necrosis factor-alpha; ESR, erythrocyte sedimentation rate. (^A^ and ^B^) level of significance within the same group by Wilcoxon test and paired *t*-test, respectively. (^C^ and ^D^) level of significance between groups using Mann–Whitney U test and unpaired *t*-test, respectively. Significance at (*p* < 0.05).

**Table 8:** Multivariable regression analysis of clinical and biochemical outcomes in the mesalamine group adjusted for demographic and disease-related confounders.

Independent Variables	β	t	*p*-Value	Dependent Variables	Significance
Age	0.016	0.041	0.9671	IBDQ-32	NS
Sex	−2.473	−0.271	0.7867		
Disease duration	7.392	1.697	0.0896		
Smoking	11.639	0.995	0.3198		
Extent: E2 vs. E1	10.114	0.856	0.3919		
Extent: E3 vs. E1	−1.542	−0.141	0.8876		
Age	−0.046	−1.270	0.2040	PMS	NS
Sex	0.347	0.462	0.6438		
Disease duration	−0.100	−0.248	0.8045		
Smoking	−0.897	−0.794	0.4273		
Extent: E2 vs. E1	0.205	0.247	0.8049		
Extent: E3 vs. E1	0.335	0.321	0.7484		
Age	−0.349	−0.413	0.6825	Calprotectin	NS
Sex	−4.386	−0.196	0.8465		
Disease duration	−0.977	−0.140	0.8895		
Smoking	−21.876	−0.929	0.3632		
Extent: E2 vs. E1	35.175	1.076	0.2924		
Extent: E3 vs. E1	−21.654	−0.427	0.6731		
Age	2.045	1.027	0.3136	TNF-α	NS
Sex	18.045	0.348	0.7302		
Disease duration	−2.899	−0.229	0.8211		
Smoking	29.423	0.851	0.4019		
Extent: E2 vs. E1	−43.162	−0.942	0.3542		
Extent: E3 vs. E1	−63.152	−1.221	0.2350		
Age	−0.053	−0.145	0.8862	ESR	NS
Sex	−0.139	−0.013	0.9898		
Disease duration	0.009	0.002	0.9985		
Smoking	−3.972	−0.456	0.6514		
Extent: E2 vs. E1	−3.832	−0.488	0.6294		
Extent: E3 vs. E1	−7.030	−0.732	0.4711		

IBDQ-32, Inflammatory Bowel Disease Questionnaire; PMS, partial Mayo score; TNF-α, tumor necrosis factor-alpha; ESR, erythrocyte sedimentation rate; NS, non-significant. E1 = proctitis, E2 = left-sided colitis, E3 = extensive colitis. Significance at (*p* < 0.05).

**Table 9:** Multivariable regression analysis of clinical and biochemical outcomes in the PTX group adjusted for demographic and disease-related confounders.

Independent Variables	β	t	*p*-Value	Dependent Variables	Significance
Age	0.551	0.641	0.5215	IBDQ-32	NS
Sex	−11.151	−0.807	0.4196		
Disease duration	−3.659	−0.938	0.3483		
Smoking	4.400	0.250	0.245		
Extent: E2 vs. E1	2.287	0.177	0.8598		
Extent: E3 vs. E1	−6.233	−0.307	0.7586		
Age	−0.040	−0.414	0.6786	PMS	NS
Sex	0.308	0.212	0.8319		
Disease duration	0.241	0.612	0.5405		
Smoking	−0.334	−0.470	0.6387		
Extent: E2 vs. E1	0.453	0.450	0.6528		
Extent: E3 vs. E1	−0.067	−0.055	0.9563		
Age	−0.750	−0.680	0.5026	Calprotectin	NS
Sex	5.999	0.207	0.8378		
Disease duration	−7.345	−0.908	0.3732		
Smoking	20.558	1.183	0.2473		
Extent: E2 vs. E1	64.958	1.706	0.0994		
Extent: E3 vs. E1	63.341	1.286	0.2094		
Age	6.020	1.686	0.1039	TNF-α	NS
Sex	−34.297	−0.395	0.6957		
Disease duration	−7.232	−0.467	0.6438		
Smoking	34.317	1.362	0.1852		
Extent: E2 vs. E1	0.626	0.010	0.9923		
Extent: E3 vs. E1	−29.739	−0.359	0.7218		
Age	0.080	0.289	0.7746	ESR	NS
Sex	−3.261	−0.536	0.5962		
Disease duration	−0.110	−0.074	0.9414		
Smoking	−4.523	−0.616	0.5433		
Extent: E2 vs. E1	3.129	0.376	0.7098		
Extent: E3 vs. E1	−1.821	−0.181	0.8581		

IBDQ-32, Inflammatory Bowel Disease Questionnaire; PMS, partial Mayo score; TNF-α, tumor necrosis factor-alpha; ESR, erythrocyte sedimentation rate; NS, non-significant. E1 = proctitis, E2 = left-sided colitis, E3 = extensive colitis. Significance at (*p* < 0.05).

**Table 10:** Unadjusted and BH-FDR-adjusted *p*-values for IBDQ subdomains and inflammatory markers.

Outcome	Unadjusted *p*-Value	BH-FDR Adjusted *p*
Systemic domain	0.0006	0.004
Calprotectin	0.003	0.012
TNF-α	0.012	0.024
Social domain	0.015	0.030
ESR	0.017	0.034
Total IBDQ-32	0.036	0.048
Digestive domain	0.036	0.048
Emotional domain	0.127	0.127

IBDQ-32, Inflammatory Bowel Disease Questionnaire; TNF-α, tumor necrosis factor-alpha; ESR, erythrocyte sedimentation rate.

**Table 11:** Analysis of drug-related side effects between the studied groups.

Side Effect	Mesalamine Group; n = 26 (%)	PTX Group; n = 27 (%)	*p*-Value
Vomiting	4 (15.38%)	3 (11.11%)	0.704
Skin rash	5 (19.2%)	4 (14.8%)	0.727
Flushing	3 (11.5%)	5 (18.5%)	0.704
Heartburn	2 (7%)	4 (14.8%)	0.668

Data are presented as numbers and percentages. Mesalamine group, UC patients treated with mesalamine and placebo; PTX group, UC patients treated with mesalamine plus pentoxifylline. Significance at (*p* < 0.05) using Fisher’s exact test.

## Data Availability

The data presented in this study are available on request from the corresponding author. The data are not publicly available due to privacy and ethical restrictions.
